# HPV E6, E6AP and cervical cancer

**DOI:** 10.1186/1471-2091-9-S1-S4

**Published:** 2008-10-21

**Authors:** Sylvie Beaudenon, Jon M Huibregtse

**Affiliations:** 1 Asuragen, Inc., 2150 Woodward, Austin, TX 78744, USA; 2 Molecular Genetics and Microbiology, Institute for Cellular and Molecular Biology, University of Texas at Austin, Austin, TX 78712, USA

## Abstract

Every year, approximately 470,000 new cases of cervical cancer are diagnosed and approximately 230,000 women worldwide die of the disease, with the majority (~80%) of these cases and deaths occurring in developing countries. Human papillomaviruses (HPVs) are the etiological agents in nearly all cases (99.7%) of cervical cancer, and the HPV E6 protein is one of two viral oncoproteins that is expressed in virtually all HPV-positive cancers. E6 hijacks a cellular ubiquitin ligase, E6AP, resulting in the ubiquitylation and degradation of the p53 tumor suppressor, as well as several other cellular proteins. While the recent introduction of prophylactic vaccines against specific HPV types offers great promise for prevention of cervical cancer, there remains a need for therapeutics. Biochemical characterization of E6 and E6AP has suggested approaches for interfering with the activities of these proteins that could be useful for this purpose.

Republished from Current BioData's Targeted Proteins database (TPdb; ).

## Protein pathway involvement in disease

### Background

Human papillomaviruses (HPVs) are small DNA tumor viruses that infect cutaneous or mucosal epithelial cells, causing papillomas or warts on skin, genital tissues and the upper promyelocytic leukemia respiratory tract [1]. More than a hundred different HPV types have been characterized, and approximately a third of these infect the genital tract [2]. The genital HPV types are sexually transmitted and can be further divided into low-risk and high-risk groups according to the propensity of their induced lesions to progress to malignancy. The low-risk HPV types, such as types 6 and 11, are associated with genital warts that do not progress toward cancer, while the high-risk HPV types cause intraepithelial lesions that can progress to invasive carcinomas [3]. High-risk HPV types are associated with 99.7% of cervical cancers [4,5]. Specifically, HPV16 and HPV18 are the high-risk types most frequently associated with cervical squamous cell carcinomas (50% and 20%, respectively). The introduction of prophylactic vaccines against specific HPV types offers great promise for prevention of these cancers [6]. High-risk HPV types are also associated with approximately 25% of head and neck carcinomas (of the mouth, tonsils, esophagus and larynx, in particular) [7,8].

The HPV E6 and E7 proteins are the only two viral genes expressed in virtually all HPV-positive cervical carcinomas, and many lines of experiments have shown that these are cooperative viral oncoproteins (reviewed in [9]). The activities of E6 and E7 that are most clearly linked to carcinogenesis are their abilities to inactivate the p53 and the retinoblastoma (pRb) tumor suppressors, respectively. High-risk HPV E6 proteins bind directly to E6AP, a cellular ubiquitin ligase encoded by the *UBE3A *gene, causing its substrate specificity to be altered so that it stably associates with and polyubiquitylates p53, resulting in degradation of p53 by the 26S proteasome [10,11]. Therefore, E6 acts (at least in part) as an oncoprotein by stimulating the destruction of perhaps the most important tumor suppressor proteins in human cancer. The E7 protein also promotes the proteasome-dependent degradation of pRb and p130 [12,13]. While the mechanism remains uncharacterized, it does not appear to involve E6AP.

**Figure 1:**
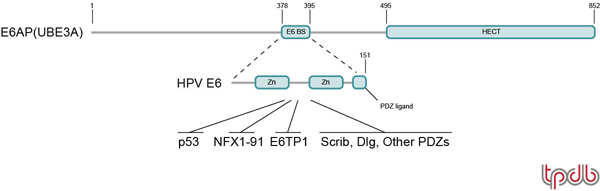
**Structural and functional domains of E6AP and E6**. The schematic of E6AP (also known as UBE3A) indicates the location of the E6 binding site (E6 BS) and the catalytic HECT domain. The two zinc binding domains and the C-terminal PDZ binding epitope of HPV16 E6 are indicated. Some of the targets that are inactivated by the E6/E6AP complex are indicated.

It is clear that high-risk HPV E6 proteins have additional, p53-independent functions, some of which could be related to cellular immortalization or transformation. For example, expression of HPV16 E6 in the skin of transgenic mice induces malignant skin tumors, and this is independent of p53 genotype [14]. E6 also induces telomerase activity [15,16], which is generally associated with cancer-derived cells. E6-induced telomerase activation requires E6AP, as demonstrated by the loss of telomerase activity following siRNA knockdown of E6AP [17,18]. This is due to targeting of a transcriptional repressor of *TERT *gene expression, NFX1-91, by the E6/E6AP complex [18]. Several other proteins are targeted by the E6/E6AP complex *in vitro *and *in vivo*, including an array of PDZ domain proteins [19-23], E6TP1 [24,25], MCM7 [26] and Bak [27] (Figure 1). While the ability of E6 to bind to PDZ domain proteins is correlated with the ability of the latter to induce epithelial hyperplasia when expressed in mouse epidermis [28], it is unclear which specific PDZ domain proteins are relevant to this activity. Finally, gene expression profiling of HPV-positive cell lines revealed that the global effects of knocking-down E6 expression by siRNAs were nearly identical to the effects of knocking-down E6AP expression, again suggesting that the complete range of E6 functions could be dependent on E6AP [17]. It should also be noted that high-risk HPVs have also been associated with head and neck cancers, particularly tonsillar carcinoma [29].

### The HPV E6 and E6AP proteins

The first *in vitro *experiments demonstrating the association of E6 with p53 and E6-induced ubiquitylation of p53 were performed in a rabbit reticulocyte lystate translation system [11,30]. Subsequent experiments showed that, in addition to ubiquitin, E1 and E2 enzymes, rabbit reticulocyte contributed a factor that was required for both stable association of E6 with p53 and p53 ubiquitylation [31]. This factor was eventually identified as E6AP [32], and by several criteria, E6AP was defined as an E3 enzyme of the ubiquitin system [10]. Primary sequence analysis of E6AP revealed an approximately 350 amino acid C-terminal domain, now known as the HECT (homologous to E6AP carboxyl-terminus) domain, which defines a large family of ubiquitin ligases [33]. There are approximately 50 HECT E3s in humans and five in *Saccharomyces cerevisiae*, and they appear to be present in all eukaryotes. The HECT E3s are unique when compared with all other known classes of E3s [34] in that, like the E1 and E2 enzymes of the ubiquitin system, they also have an active-site cysteine (within the HECT domain) that forms a ubiquitin thioester intermediate during their reaction cycle [35].

**Figure 2:**
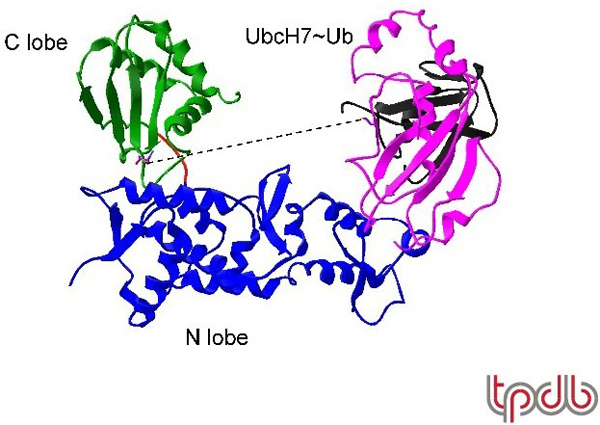
**The X-ray crystal structure of the complex of the E6AP HECT domain with UbcH7 (PDB 1DF5)**. The HECT N lobe (blue), linker (red) and C lobe (green) are indicated. UbcH7 (magenta) was co-crystallized with the HECT domain, while ubiquitin (black) is modeled into the structure shown based on a Ubc1-ubiquitin model [72] (PDB 1FXT). The dashed line represents the 41 Å line-of-site between the active-site cysteines of UbcH7 and E6AP. Adapted from [38].

X-ray crystal structures of the HECT domain of three different HECT E3s (E6AP, WWP1 and Smurf2) are now available [36-38]. The domain consists of a large N-terminal lobe (approximately 250 amino acids) and, connected by a short flexible hinge, a C-terminal lobe of approximately 100 amino acids, which contains the active-site cysteine (Figure 2). The E6AP-UbcH7 structure (2.6 Å resolution) revealed the basis for interaction of the HECT domain with the activating E2 protein, which docks onto a surface of the N-terminal lobe [38]. The affinity of the E2 for the HECT domain is quite low (approximately 6 μM) [39], but this appears to be advantageous for release of the E2 so that it can be re-charged following transfer of ubiquitin to the HECT active site [39]. Binding and release appears to be required for catalysis of polyubiquitylation since the surface of the E2 that interacts with the HECT domain is predicted to overlap with the surface that interacts with the E1 enzyme.

The most puzzling aspect of the E6AP-UbcH7 structure was the large distance separating the active-site cysteines of the two proteins; approximately 41 Å [38]. This distance obviously precludes a direct transthiolation reaction without a large conformational rearrangement. It was therefore interesting to observe that in the WWP1 structure [37], the active sites were predicted to be approximately 16 Å apart (after modeling in the E2 structure based on the E6AP-UbcH7 structure). This difference was due largely to a conformational change at the hinge connecting the N- and C-terminal lobes. In the Smurf2 structure, the cysteines were predicted to be even further separated than in the E6AP structure (50 Å) [36]. These alternative structures have led to the hypothesis that conformational changes about the flexible hinge accompany the transthiolation and substrate ubiquitylation reactions, with the three known structures perhaps representing intermediates that each of these proteins might pass through during the reaction cycle. It appears unlikely that E2 binding initiates the conformational changes, since the conformation of E6AP was essentially identical with or without UbcH7 [38]. Also unresolved is the mechanism of polyubiquitylation and the basis for synthesis of different chain types (e.g. Lys48 versus Lys63 polyubiquitylation). For example, E6AP has a strong preference for assembly of Lys48-linked polyubiquitin chains, while *S. cerevisiae *Rsp5 has a strong preference for assembly of Lys63-linked chains [40,41]. Given the very different outcomes of Lys48 and Lys63 polyubiquitylation [42], it is important to determine the mechanistic distinction between Lys48- and Lys63-specific HECT E3s.

The HECT domain, by itself, contains all the determinants necessary for formation of a ubiquitin thioester intermediate [43,44], suggesting that it is a self-contained catalytic domain. Since all HECT E3s are large proteins (ranging in size from 92 to over 500 kDa), it has been proposed that the divergent sequences upstream of the HECT domain contain the determinants of substrate specificity [33]. This model is supported by characterization of the E6/E6AP complex, where the E6 binding domain is localized to an 18 amino acid alpha helical epitope in the central portion of the protein, centered approximately 120 amino acids upstream of the HECT domain [32,45,46]. Deletion or specific point mutations within this helix completely abrogate both E6 and p53 association, and ubiquitylation of p53 *in vitro *[47]. Consistent with this 'two domain' model, whereby substrates are recognized by N-terminal epitopes and ubiquitylated by the C-terminal HECT domain, the WW domains found in the central region of a subgroup of HECT E3s (e.g. yeast Rsp5 and human WWP1 and Nedd4) have been shown in many cases to be the primary determinants of substrate specificity of these enzymes (reviewed in [48]). Structure-function relationships of E6AP and E6 are summarized in Figure 1.

Based on the notion that E6 redirects the substrate specificity of E6AP, a simple model is that E6 binds to both E6AP and p53, bridging the interaction between enzyme and substrate. However, while E6 binds directly to E6AP, *in vitro *binding assays indicate that neither E6 nor E6AP interacts significantly with p53 in the absence of the other [32]. This suggests that either the two proteins together form a surface for p53 interaction, or that one of these proteins influences the conformation of the other so that it can interact with p53. It should be noted that there have been reports that E6 does bind directly to p53 in the absence of E6AP [49], however the significance of this interaction is contentious [50]. In this context, the basis for variable findings could be due to the problems in expressing purified soluble E6 protein for biochemical experiments. The bridging model clearly applies to the interaction of the E6/E6AP complex with its PDZ domain protein targets. E6 is able to bind directly to these target proteins, both independently of E6AP as well as simultaneously to both PDZ domain proteins and E6AP [22]. The interaction between E6 and PDZ domain proteins is mediated by a conserved C-terminal PDZ binding epitope (consisting of S/T-x-V/L). Interestingly, all of the high-risk cancer-associated HPV E6 proteins contain a consensus PDZ binding epitope that is absent in all other papillomavirus E6 proteins. While this is compelling circumstantial evidence that targeting of PDZ proteins could play a role in carcinogenesis, this has not yet been directly demonstrated.

The E6 proteins have been very difficult to analyze structurally, even though they are only 150–160 amino acids in length. This is due to aggregation when they are expressed in significant amounts [51]. All E6 proteins contain two zinc binding domains, each consisting of a C-X-X-C-X_29_-C-X-X-C sequence (Figure 1). An NMR structure of the C-terminal zinc binding domain of HPV16 E6 was recently solved and a model of the full-length protein was proposed, suggesting that E6 proteins consist of a conserved structural scaffold with highly variable surfaces participating in specialized HPV functions [52]. While many studies have characterized E6 mutants with respect to E6 and p53 association, there is no structural information that indicates which surface of E6 interacts with either E6AP or p53.

In summary, the interaction of E6 with E6AP directs the ubiquitylation activity of E6AP toward several specific cellular proteins, the most notable of which with respect to carcinogenesis is p53. Surprisingly, very few of the natural (i.e. E6-independent) targets of E6AP have been identified. This is a very important problem, since mutations or disruption in expression of E6AP in the brain are the cause of Angelman syndrome (AS), a severe form of mental retardation [53-55]. Though E6AP is expressed in virtually all cells of the human body, in subregions of the brain (including the hippocampal neurons and Purkinje cells) E6AP is expressed only from the maternal allele. AS is caused by disruptions in expression of the maternal allele of E6AP, generally by large chromosomal deletion but also by point mutations within the E6AP coding sequence. This strongly suggests that lack of ubiquitylation of one or more E6AP target proteins in the brain is responsible for the severe AS phenotype.

## Disease models, knockouts, assays

While the discovery of the Shope cottontail rabbit papillomavirus (CRPV) was one of the earliest descriptions of an animal DNA tumor virus [56], there are no animal models for cervical carcinogenesis involving the high-risk HPVs. Moreover, though a rhesus monkey sexually transmitted papillomavirus has been associated with malignancies [57], it has not been established that the E6 protein of this virus (RhPV-1) targets p53 for degradation or interacts with E6AP. RhPV-1 E6 clusters with the high-risk HPV E6 proteins based on sequence similarity, although it lacks their characteristic PDZ binding epitope.

The laboratory of Paul Lambert (University of Wisconsin) developed an E6 transgenic mouse model that has been very useful in dissecting the functions of E6 in a multi-step model of skin cancer [14]. Transgenic mice expressing a mutant of E6 defective for E6AP interaction showed markedly reduced phenotypes associated with E6 expression, including the development of spontaneous skin tumors [58]. In addition, the PDZ binding activity of E6 was found to contribute to the progression stage of carcinogenesis in this model, but not to the promotion stage [59]. The PDZ binding activity was also important for the ability of E6 to induce epithelial hyperplasia when expressed in the eye [28]. Therefore, the transgenic mouse system, combined with the knowledge from biochemical characterization of E6 and E6AP, has proven to be a useful tool for dissecting the contribution of various E6 activities to carcinogenesis.

The productive replication cycle of papillomaviruses is notoriously difficult to study given the tight linkage of virus transcription and replication to the differentiation program of the infected epithelial tissue. The most useful system for studying this in the laboratory has been the organotypic raft culture system, where cultured primary keratinocytes can be induced to differentiate at the air-liquid interface. Laimins and colleagues (Northwestern University) have shown that, in this context, cells that contain HPV DNA will yield infectious papillomavirus particles [60]. By introducing specific mutations into different viral ORFs, this system allows some very basic questions to be addressed [60,61]. In this system, deletion of the PDZ binding domain of HPV31 E6 had relatively little effect on virus replication, although there was a slight effect on viral DNA copy number and early stimulation of proliferation [62]. The use of cells suspended in semi-solid media (methylcellulose) is another way in which differentiation can be recapitulated in a manner that supports viral propagation [63]. The contribution of E6AP to virus replication has, to our knowledge, not been directly examined in these systems to date.

Finally, it should be emphasized that several HPV-positive cell lines derived from cervical cancers or their metastases are widely available, and these are useful for addressing certain HPV E6 functions, including targeting of p53. The best known HPV-containing cell line is HeLa, an HPV18-containing cell line isolated over 50 years ago from a cervical carcinoma patient [64]. Perhaps surprisingly, even after 50 years in culture, HeLa cells are still dependent on continued expression of E6 for their survival, as knockdown of either E6 or E6AP with siRNA results in the rapid accumulation of p53 [17] and the induction of apoptosis [65]. Other commonly used HPV cell lines available include SiHa (HPV16), Caski (HPV16) and C4-I (HPV18).

## Disease targets and ligands

Our knowledge of the molecular details of the interactions between E6 and E6AP, as well as between E6AP and activating E2 enzymes, present opportunities for interfering with degradation of p53 in HPV-positive cervical cancers or precursor lesions. The NMR structure of the 18 amino acid E6 binding domain of E6AP has been determined [45]. The leucine residues within this peptide form a hydrophobic patch on a face of the helix, and mutation of any of these residues to alanine abolishes E6 binding [45]. Recently, building on the structural and mutagenesis information, a three-dimensional query was developed by Baleja and co-workers (Tufts University School of Medicine) to identify potential small molecule inhibitors of E6 binding [66]. This approach successfully identified compounds that blocked E6 binding and stabilized p53 in E6-expressing cells. These compounds represent exciting lead molecules for future development. Other attempts at inhibiting E6 function include work by Beerheide and co-workers (Institute of Molecular and Cell Biology, Singapore) identifying compounds that chelate zinc away from E6 [67,68].

The HECT domain-E2 interaction surface represents another potential drug target. Since the surface of the E2 that interacts with the HECT domain is involved in interactions with many E3s, as well as with the E1 enzyme, targeting of the surface of the HECT domain could provide greater specificity. The molecular details of this interaction are well understood [38,39]. Another route toward inhibiting E6AP would be to isolate compounds that block the large conformational rearrangements that are presumed to occur during the reaction cycle. The C-terminal tail of the HECT domain contains a conserved phenylalanine residue (the -4F, named for its location relative to the C-terminus), and mutation of this reside blocks protein ubiquitylation, but not ubiquitin thioester formation [69]. The tail residues could therefore also be attractive drug targets for blocking protein ubiquitylation.

## New frontiers in drug discovery

The future is certain to see the continued development of more advanced prophylactic HPV vaccines that target a wider variety of HPV types, as well as therapeutic HPV vaccines. Nevertheless, effective antiviral compounds could be useful in treatment of pre-existing disease. E6 is an excellent target because it is expressed through all stages of carcinogenesis and its mode of action is well characterized. In addition, established *in vitro *assays and cell-based assays for E6/E6AP-dependent ubiquitylation of p53 present excellent opportunities for isolation of compounds that inhibit specific protein-protein associations or specific steps in the catalytic cycle.

A concern about any approach aimed at inhibiting E6AP is that loss-of-function mutations in E6AP/*UBE3A *cause AS. The natural, E6-independent targets of E6AP are poorly characterized, although one interesting target recently reported is the Rho-GEF Pbl/Ect2, which is regulated by mouse E6AP in neurons [70]. While it is not known whether targeting of Pbl/Ect2 is related to AS, the effects of blocking the ubiquitylation of the complete set of E6-independent targets of E6AP must be considered during attempts to interfere with E6AP activity in HPV-induced lesions. This consideration could rule out systemic delivery of anti-E6AP drugs, although the nature of HPV infections suggests that topical drug application might, at least in some cases, be a viable delivery route. Finally, further biochemical and mechanistic studies of HECT E3s could reveal novel approaches for interfering with specific aspects of the reaction cycle. For example, E6AP catalyzes the formation of Lys48-linked polyubiquitin chains, while some other HECT E3s, such as yeast Rsp5, have a strong preference for catalysis of Lys63-linked chains [40,41]. Lys48 chains typically target proteins to the proteasome, while it is clear that Lys63-linked chains can have non-proteolytic functions [71]. Therefore, inhibition or alteration of the type of polyubiquitin chain synthesized by E6AP could be a viable target for drug discovery.

## List of abbreviations used

AS: Angelman syndrome; CRPV: cottontail rabbit papillomavirus; E6AP: E6-associated protein; HECT: homologous to E6AP carboxyl-terminus; HPV: human papillomavirus; ORF: open reading frame; RhPV: rhesus monkey papillomavirus.

## Competing interests

The authors declare that they have no competing interests.

## Publication history

Republished from Current BioData's Targeted Proteins database (TPdb; ).
